# First determination and analysis of the complete mitochondrial genome of X-ray tetra *Pristella maxillaris* (Ulrey, 1894) (Actinopteri, Characidae)

**DOI:** 10.1080/23802359.2022.2026263

**Published:** 2022-01-24

**Authors:** Chuangbin Tang, Lijuan Wei, Qiuchan Huang, Qihai Zhou, Guohai Wang

**Affiliations:** a College of Chemistry and Bioengineering, Guangxi Normal University for Nationalities, Chongzuo, China; b Key Laboratory of Ecology of Rare and Endangered Species and Environmental Protection, Ministry of Education, Guangxi Normal University, Guilin, China; c College of Physics and Electronic Engineering, Guangxi Normal University for Nationalities, Chongzuo, China

**Keywords:** X-ray tetra, mitochondrial genome, Characidae, *Pristella maxillaris*

## Abstract

The complete mitochondrial genome of the X-ray tetra (*Pristella maxillaris*, Ulrey, 1894) was determined by using next-generation sequencing technology, and its mitochondrial genome characteristics were analyzed. The sequence total length was 16,753 bp, and the A + T content was 57.44%. The position and composition of the 37 genes were consistent with those of other Characidae species in this family. There are 13 protein-coding genes, 22 tRNA genes, two rRNA genes, and one control region. Except for *ND2* and *COX1*, which use ATT and GTG as start codons, respectively, all other protein-coding genes use ATG as the start codon. *COX1* uses AGG as the stop codon; *ATP6* and *COX3* use incomplete TA as the stop codon; *COX2* and *ND4* use incomplete T as the stop codon; *ATP8* uses TAG as the stop codon, and the other seven protein-coding genes use TAA as the stop codon. Based on the concatenated nucleotide sequences of 13 protein-coding genes from 18 Characidae species, phylogenetic analysis revealed that *P. maxillaris* belongs to the family Characidae and is most closely related to *Hyphessobrycon amandae.* Determining the mitogenomes of *P. maxillaris* improves our understanding of the phylogeny and evolution of Characidae.

Ornamental fish refers to fish with bright colors or strange shapes with ornamental value (Sun et al. 2021). We choose one of the most kept fish – the X-ray tetra (*Pristella maxillaris*, Ulrey, 1894) as the research object. The X-ray tetra belongs to the order Characiformes and family Characidae. It is mainly distributed in South America in the Amazon, Orinoco, and coastal river drainages of the Guianas (Boujard et al. 1997). We amplified and measured the mtDNA of *P. maxillaris*. The sequence structure was predicted and analyzed based on the splicing and annotation of the sequence. These results can be used for systematic classification, germplasm resource protection, and population genetic diversity analysis of *P. maxillaris* and other Characidae fishes (Bundesministerium für Ernährung, Landwirtschaft und Forsten (BMELF) 1999; Mills and Vevers 1989; Planquette et al. 1996; Weitzman and Palmer 1997).

The complete mitochondrial genome of *P. maxillaris* was determined by next-generation sequencing. Specimens were collected from Jiangzhou District, Chongzuo City, Guangxi (107°23′28.24″ E, 22°23′13.97″ N). The collection and sampling of the specimens were reviewed and approved by the Animal Ethics Committee of Guangxi Normal University for Nationalities. Voucher specimens were deposited at the Institute of Chemistry and Bioengineering, Guangxi Normal University for Nationalities, China (Guohai Wang, 1016729581@qq.com), and the voucher number was CCB-202006. After collection, total genomic DNA was isolated by using a universal genomic DNA extraction kit (Beijing, China) and sequenced on an Illumina HiSeq sequencer (Illumina, Inc., San Diego, CA, USA) using the PE150 protocol. The raw reads were filtered by using Trimmomatic and assembled by using GetOrganelle (Jin et al. 2018).

The total length of the *P. maxillaris* mitochondrial genome was 16,753 bp. According to the annotation results from the NCBI Mitochondrial Database, the genome contains 13 protein-coding genes, 22 tRNA genes, 2 rRNA genes, and one control region. The nucleotide composition of this mitogenome was 29.40% A, 26.63% C, 15.92% G, and 28.04% T, with a higher A + T content (57%) than the G + C content (42%). The nucleotide composition and AT content of the entire mitogenome were similar to those of other vertebrate mitogenomes (Sun et al. 2020a, 2020b).

The phylogenetic relationships of *P. maxillaris* with Characidae species (Sun et al. 2021) were reconstructed based on the concatenated sequences of 13 protein-coding genes using maximum-likelihood analysis, and three fish species (*Salminus brasiliensis*, *Lateolabrax japonicus*, and *Piaractus brachypomus*) were selected to root the tree (Figure 1). *P. maxillaris* was recovered as the sister group of *Hyphessobrycon amandae* with maximal support, thus validating the close relationship between the two species. This clade was grouped with *Gephyrocharax atracaudatus*, and *Hasemania nana* was the sister to the clade containing *P. maxillaris, G. atracaudatus* and *Hyphessobrycon amandae*.

**Figure 1.**
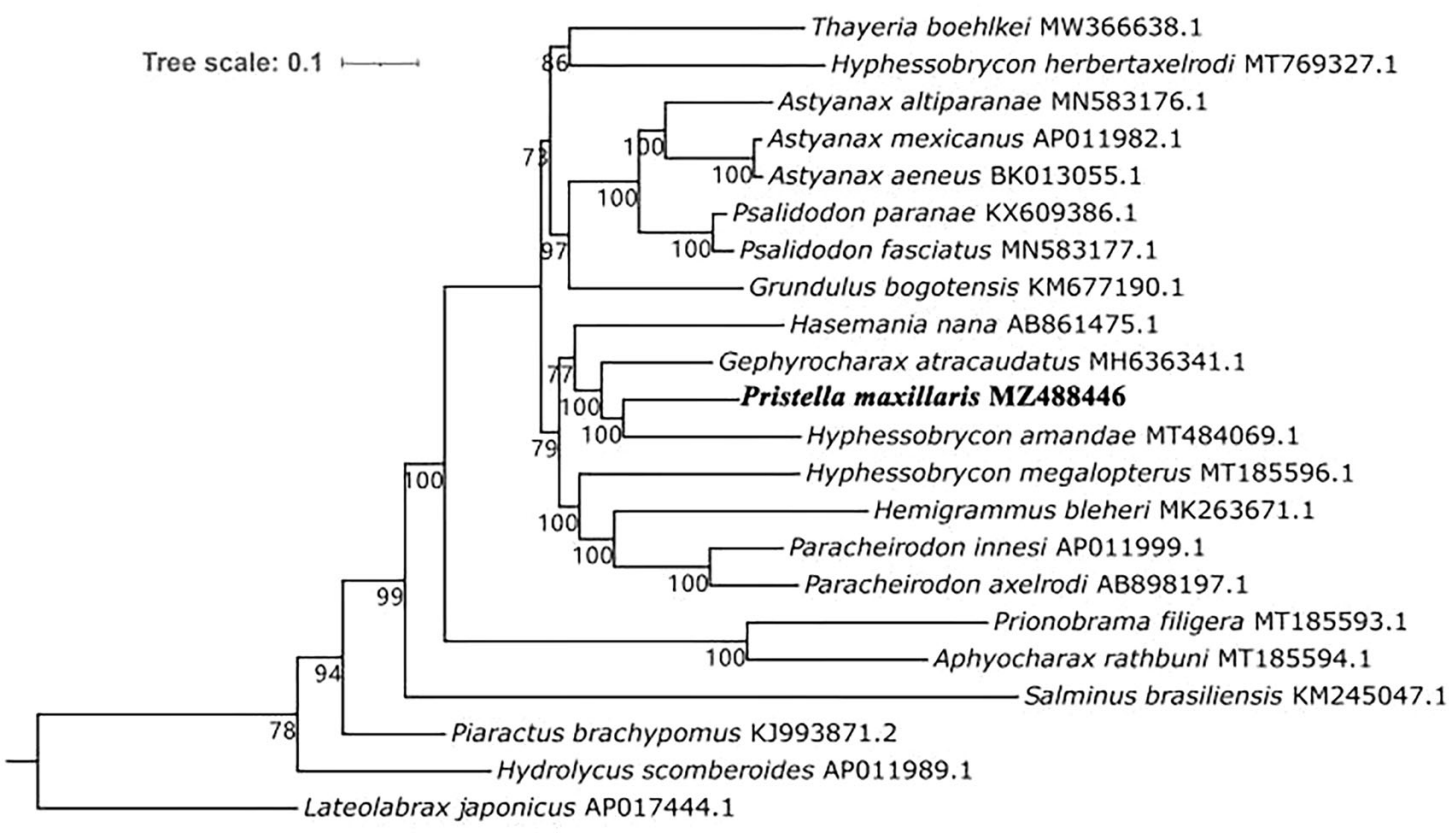
Maximum-likelihood phylogenetic relationships of Crassostrea based on the concatenated sequence of 13 protein-coding genes using IQ-TREE v1.6.8. The *Lateolabrax japonicus* was used as outgroup. The *P. maxillaris* genome was marked in bold font.

## Data Availability

The data that support the findings of this study are available in the NCBI GenBank database at https://www.ncbi.nlm.nih.gov/, reference number MZ488446. The associated BioProject, BioSample, and SRA numbers are PRJNA742850, SAMN19981405, SRR15012162, respectively (https://www.ncbi.nlm.nih.gov/sra/?term=SRR15012162).

## References

[CIT0001] Boujard T, Pascal M, Meunier FJ, Le Bail PY. 1997. Poissons de Guyane. Guide écologique de l'Approuague et de la réserve des Nouragues. Paris: Institut National de la Recherche Agronomique; p. 219.

[CIT0002] Bundesministerium für Ernährung, Landwirtschaft und Forsten (BMELF). 1999. Gutachten über Mindestanforderungen an die Haltung von Zierfischen (Süßwasser). Bonn, Germany: Bundesministerium für Ernährung, Landwirtschaft und Forsten (BMELF); p. 16.

[CIT0003] Jin JJ, Yu WB, Yang JB. 2018. GetOrganelle: a simple and fast pipeline for de novo assembly of a complete circular chloroplast genome using genome skimming data. BioRxiv. 2018:256479.

[CIT0004] Mills D, Vevers G. 1989. The Tetra encyclopedia of freshwater tropical aquarium fishes. New Jersey: Tetra Press; p. 208.

[CIT0005] Planquette P, Keith P, Bail Py L. 1996. Atlas des poissons d'eau douce de Guyane. Tome 1. Collection du Patrimoine Naturel. Volume 22. Paris: MNHN, Paris & INRA; p. 429.

[CIT0006] Sun CH, Liu HY, Lu CH. 2020a. Five new mitogenomes of *Phylloscopus* (Passeriformes, Phylloscopidae): sequence, structure, and phylogenetic analyses. Int J Biol Macromol. 146:638–647. 31899244 10.1016/j.ijbiomac.2019.12.253

[CIT0007] Sun CH, Liu HY, Min X, Lu CH. 2020b. Mitogenome of the little owl Athene noctua and phylogenetic analysis of Strigidae. Int J Biol Macromol. 151:924–931. 32097733 10.1016/j.ijbiomac.2020.02.238

[CIT0008] Sun CH, Liu HY, Xu N, Zhang XL, Zhang Q, Han BP. 2021. Mitochondrial genome structures and phylogenetic analyses of two tropical Characidae fishes. Front Genet. 12:627402. 33633787 10.3389/fgene.2021.627402 PMC7901900

[CIT0009] Weitzman SH, Palmer L. 1997. A new species of *Hyphessobrycon* (Teleostei: Characidae) from Neblina region of Venezuela and Brazil, with comments on the putative 'rosy tetra clade. Ichthyol. Explor. Freshwat. 7(3–4):209–242.

